# Characteristics and Maternal and Birth Outcomes of Hospitalized Pregnant Women with Laboratory-Confirmed COVID-19 — COVID-NET, 13 States, March 1–August 22, 2020

**DOI:** 10.15585/mmwr.mm6938e1

**Published:** 2020-09-25

**Authors:** Miranda J. Delahoy, Michael Whitaker, Alissa O’Halloran, Shua J. Chai, Pam Daily Kirley, Nisha Alden, Breanna Kawasaki, James Meek, Kimberly Yousey-Hindes, Evan J. Anderson, Kyle P. Openo, Maya L. Monroe, Patricia A. Ryan, Kimberly Fox, Sue Kim, Ruth Lynfield, Samantha Siebman, Sarah Shrum Davis, Daniel M. Sosin, Grant Barney, Alison Muse, Nancy M. Bennett, Christina B. Felsen, Laurie M. Billing, Jessica Shiltz, Melissa Sutton, Nicole West, William Schaffner, H. Keipp Talbot, Andrea George, Melanie Spencer, Sascha Ellington, Romeo R. Galang, Suzanne M. Gilboa, Van T. Tong, Alexandra Piasecki, Lynnette Brammer, Alicia M. Fry, Aron J. Hall, Jonathan M. Wortham, Lindsay Kim, Shikha Garg, COVID-NET Surveillance Team, COVID-NET Surveillance Team, Mirasol Apostol, Susan Brooks, Ashley Coates, Linda Frank, Brooke Heidenga, Kareena Hundal, Joelle Nadle, Sherry Quach, Jeremy Roland, Maria Rosales, Isaac Armistead, Rachel Herlihy, Sarah McLafferty, Adam Misiorski, Christina Parisi, Danyel Olson, Carol Lyons, Amber Maslar, Paula Clogher, David Blythe, Alicia Brooks, Rachel Park, Michelle Wilson, Erica Bye, Kathryn Como-Sabetti, Richard Danila, Maureen Sullivan, Kathy M. Angeles, Melissa Christian, Nancy Eisenberg, Caroline Habrun, Emily B. Hancock, Sarah A. Khanlian, Meaghan Novi, Yadira Salazar-Sanchez, Elizabeth Dufort, Nancy Spina, Ama Owusu-Dommey, Tiffanie Markus, Ryan Chatelain, Laine McCullough, Jake Ortega, Andrea Price, Ashley Swain, Anita Kambhampati, Seth Meador

**Affiliations:** ^1^CDC COVID-NET Team; ^2^Epidemic Intelligence Service, CDC; ^3^Eagle Global Scientific, Atlanta, Georgia; ^4^California Emerging Infections Program, Oakland California; ^5^Colorado Department of Public Health and Environment; ^6^Connecticut Emerging Infections Program, Yale School of Public Health, New Haven, Connecticut; ^7^Departments of Pediatrics and Medicine, Emory University School of Medicine, Atlanta, Georgia; ^8^Emerging Infections Program, Atlanta Veterans Affairs Medical Center, Atlanta, Georgia; ^9^Foundation for Atlanta Veterans Education and Research, Decatur, Georgia; ^10^Maryland Department of Health; ^11^Michigan Department of Health and Human Services; ^12^Minnesota Department of Health; ^13^New Mexico Emerging Infections Program, Albuquerque, New Mexico; ^14^New Mexico Department of Health; ^15^New York State Department of Health; ^16^University of Rochester School of Medicine and Dentistry, Rochester, New York; ^17^Ohio Department of Health; ^18^Public Health Division, Oregon Health Authority, Portland, Oregon; ^19^Vanderbilt University Medical Center, Nashville, Tennessee; ^20^Salt Lake County Health Department, Salt Lake City, Utah; ^21^Cherokee Nation Assurance, Arlington, Virginia.; California Emerging Infections Program; California Emerging Infections Program; California Emerging Infections Program; California Emerging Infections Program; California Emerging Infections Program; California Emerging Infections Program; California Emerging Infections Program; California Emerging Infections Program; California Emerging Infections Program; California Emerging Infections Program; Colorado Department of Public Health and Environment; Colorado Department of Public Health and Environment; Colorado Department of Public Health and Environment; Connecticut Emerging Infections Program, Yale School of Public Health; Connecticut Emerging Infections Program, Yale School of Public Health; Connecticut Emerging Infections Program, Yale School of Public Health; Connecticut Emerging Infections Program, Yale School of Public Health; Connecticut Emerging Infections Program, Yale School of Public Health; Connecticut Emerging Infections Program, Yale School of Public Health; Maryland Department of Health; Maryland Department of Health; Maryland Emerging Infections Program, Johns Hopkins Bloomberg School of Public Health; Maryland Emerging Infections Program, Johns Hopkins Bloomberg School of Public Health; Minnesota Department of Health; Minnesota Department of Health; Minnesota Department of Health; Minnesota Department of Health; New Mexico Emerging Infections Program; New Mexico Emerging Infections Program; New Mexico Emerging Infections Program; New Mexico Emerging Infections Program; New Mexico Emerging Infections Program; New Mexico Emerging Infections Program; New Mexico Emerging Infections Program; New Mexico Emerging Infections Program; New York State Department of Health; New York State Department of Health; Public Health Division, Oregon Health Authority; Vanderbilt University Medical Center; Salt Lake County Health Department; Salt Lake County Health Department; Salt Lake County Health Department; Salt Lake County Health Department; Salt Lake County Health Department; CDC COVID-NET Team, Cherokee Nation Assurance; CDC COVID-NET Team.

*On September 16, 2020, this report was posted online as an *MMWR *Early Release.*

Pregnant women might be at increased risk for severe coronavirus disease 2019 (COVID-19) (*1*,*2*). The COVID-19-Associated Hospitalization Surveillance Network (COVID-NET) (*3*) collects data on hospitalized pregnant women with laboratory-confirmed SARS-CoV-2, the virus that causes COVID-19; to date, such data have been limited. During March 1–August 22, 2020, approximately one in four hospitalized women aged 15–49 years with COVID-19 was pregnant. Among 598 hospitalized pregnant women with COVID-19, 54.5% were asymptomatic at admission. Among 272 pregnant women with COVID-19 who were symptomatic at hospital admission, 16.2% were admitted to an intensive care unit (ICU), and 8.5% required invasive mechanical ventilation. During COVID-19–associated hospitalizations, 448 of 458 (97.8%) completed pregnancies resulted in a live birth and 10 (2.2%) resulted in a pregnancy loss. Testing policies based on the presence of symptoms might miss COVID-19 infections during pregnancy. Surveillance of pregnant women with COVID-19, including those with asymptomatic infections, is important to understand the short- and long-term consequences of COVID-19 for mothers and newborns. Identifying COVID-19 in women during birth hospitalizations is important to guide preventive measures to protect pregnant women, parents, newborns, other patients, and hospital personnel. Pregnant women and health care providers should be made aware of the potential risks for severe COVID-19 illness, adverse pregnancy outcomes, and ways to prevent infection.

COVID-NET conducts population-based surveillance for laboratory-confirmed COVID-19–associated hospitalizations in 14 states encompassing 99 counties* (*3*). Thirteen states (California, Colorado, Connecticut, Georgia, Maryland, Michigan, Minnesota, New Mexico, New York, Ohio, Oregon, Tennessee, and Utah) contributed data to this report. Residents of the predefined surveillance catchment area who had a positive molecular test for SARS-CoV-2 during hospitalization or up to 14 days before hospital admission were classified as having a COVID-19–associated hospitalization and were included in COVID-NET surveillance. Persons included in COVID-NET surveillance are referred to as having COVID-19 throughout this report. SARS-CoV-2 testing was performed at the discretion of health care providers or through facility policies dictating uniform or criteria-based testing of patients upon admission. Trained surveillance officers performed medical chart abstractions for a convenience sample of hospitalizations using a standardized case report form. This analysis included women aged 15–49 years who were pregnant at hospital admission. Descriptive statistics were calculated for hospitalized pregnant women with complete chart review and discharge disposition (i.e., discharged or died during hospitalization). Women with one or more signs or symptoms included on the COVID-NET case report form (*3*) at the time of hospital admission were classified as symptomatic. Birth outcomes were described for pregnancies completed during a COVID-19–associated hospitalization. Reason for hospital admission was collected starting in June. Data were analyzed using SAS software (version 9.4; SAS Institute). This activity was reviewed by CDC and was conducted consistent with applicable federal law and CDC policy.^†^ Sites obtained approval for COVID-NET surveillance from their state and local institutional review boards, as required.

During March 1–August 22, 2020, COVID-NET identified 7,895 hospitalized women aged 15–49 years with COVID-19; discharge disposition was determined, and chart review was completed for 2,318 (29.4%) (Figure 1). Among 2,255 (97.3%) women with information about pregnancy status, 598 (26.5%) were pregnant, with median age 29 years. Among 577 (96.5%) pregnant women with reported race and ethnicity, 42.5% were Hispanic or Latino (Hispanic), and 26.5% were non-Hispanic Black (Black) (Table).

**FIGURE 1:**
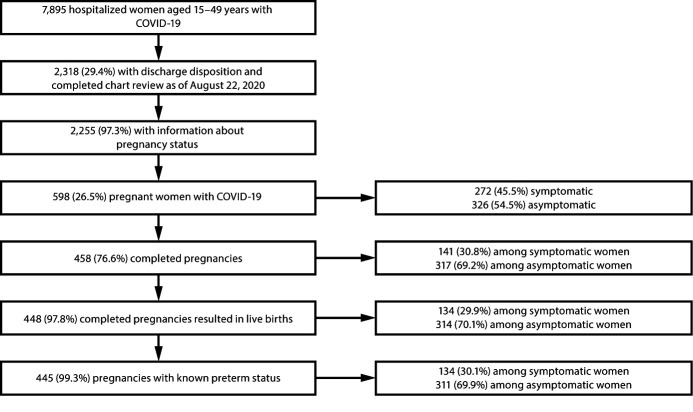
Pregnancy status, signs and symptoms,* and birth outcomes^†^^,^^§^^,^^¶^ among hospitalized women aged 15–49 years with COVID-19** — COVID-NET, 13 States,^††^ March 1–August 22, 2020 **Abbreviations:** COVID-19 = coronavirus disease 2019; COVID-NET = COVID-19-Associated Hospitalization Surveillance Network. * Symptomatic women were those who had one or more signs or symptoms (fever/chills, cough, shortness of breath, muscle aches, nausea/vomiting, headache, sore throat, abdominal pain, chest pain, nasal congestion/rhinorrhea, decreased smell, decreased taste, diarrhea, upper respiratory illness/influenza-like illness, wheezing, hemoptysis/bloody sputum, conjunctivitis, rash, altered mental state, and seizure) at hospital admission; asymptomatic women did not have any of these signs or symptoms at admission. ^†^ The 448 pregnancies resulting in live births resulted in the birth of 457 newborns; nine women had twins. Two newborns included in this category who were born alive subsequently died during the birth hospitalization. ^§^ Ten completed pregnancies resulted in pregnancy losses. Pregnancy losses might include spontaneous abortion/miscarriage, therapeutic abortion, or stillbirth. ^¶^ Pregnancies with known preterm status were those resulting in a live birth for which the gestational age at delivery was known. For three pregnancies resulting in live births, the gestational age at the time of birth was unknown. ** Women residing in the predefined COVID-NET surveillance catchment with a positive real-time reverse transcription–polymerase chain reaction (RT-PCR) test for SARS-CoV-2, during hospitalization or up to 14 days before admission. Among the 597 (99.8%) pregnant women for whom the COVID-19 test type was known, all had a positive RT-PCR test result; the COVID-19 test type for one pregnant woman with a positive COVID-19 test result was unknown. ^††^ California, Colorado, Connecticut, Georgia, Maryland, Michigan, Minnesota, New Mexico, New York, Ohio, Oregon, Tennessee, and Utah.

**TABLE:** Characteristics and outcomes of hospitalized pregnant women with COVID-19 — COVID-NET, 13 states,* March 1–August 22, 2020

Characteristic	no./No. (%)		
	Overall (N = 598)	Symptomatic at admission (n = 272)	Asymptomatic at admission (n = 326)
**Age group, yrs**			
15–24	167/598 (27.9)	69/272 (25.4)	98/326 (30.1)
25–34	318/598 (53.2)	143/272 (52.6)	175/326 (53.7)
35–49	113/598 (18.9)	60/272 (22.1)	53/326 (16.3)
**Race/Ethnicity (n = 577)**			
Hispanic or Latino	245/577 (42.5)	131/265 (49.4)	114/312 (36.5)
American Indian or Alaska Native, non-Hispanic	8/577 (1.4)	4/265 (1.5)	4/312 (1.3)
Asian or Pacific Islander, non-Hispanic	72/577 (12.5)	37/265 (14.0)	35/312 (11.2)
Black, non-Hispanic	153/577 (26.5)	57/265 (21.5)	96/312 (30.8)
White, non-Hispanic	97/577 (16.8)	35/265 (13.2)	62/312 (19.9)
Multiracial	2/577 (0.3)	1/265 (0.4)	1/312 (0.3)
**Pregnancy trimester at hospital admission (n = 596)**			
First	14/596 (2.3)	13/271 (4.8)	1/325 (0.3)
Second	61/596 (10.2)	50/271 (18.5)	11/325 (3.4)
Third	521/596 (87.4)	208/271 (76.8)	313/325 (96.3)
**Reason for hospital admission (n = 324)^†^**			
COVID-19–related illness	61/324 (18.8)	59/122 (48.4)	2/202 (1.0)
Obstetrics/Labor and delivery	242/324 (74.7)	55/122 (45.1)	187/202 (92.6)
Other	21/324 (6.5)	8/122 (6.6)	13/202 (6.4)
**Underlying conditions**			
Any underlying condition or conditions	123/598 (20.6)	63/272 (23.2)	60/326 (18.4)
Asthma	49/598 (8.2)	30/272 (11.0)	19/326 (5.8)
Cardiovascular disease (excludes hypertension)	6/598 (1.0)	6/272 (2.2)	0/326 (—)
Chronic lung disease	6/598 (1.0)	6/272 (2.2)	0/326 (—)
Chronic metabolic disease	44/598 (7.4)	23/272 (8.5)	21/326 (6.4)
Diabetes mellitus^§^	23/598 (3.8)	15/272 (5.5)	8/326 (2.5)
Thyroid dysfunction	21/598 (3.5)	9/272 (3.3)	12/326 (3.7)
Hypertension	26/598 (4.3)	12/272 (4.4)	14/326 (4.3)
Liver disease	10/598 (1.7)	5/272 (1.8)	5/326 (1.5)
Neurologic conditions	12/598 (2.0)	6/272 (2.2)	6/326 (1.8)
Other underlying condition or conditions^¶^	7/598 (1.2)	3/272 (1.1)	4/326 (1.2)
**Smoking**			
Current smoker	13/598 (2.2)	8/272 (2.9)	5/326 (1.5)
Former smoker	41/598 (6.9)	20/272 (7.4)	21/326 (6.4)
Not a smoker/Unknown smoking history	544/598 (91.0)	244/272 (89.7)	300/326 (92.0)
Chest radiograph findings (n = 132)**			
Infiltrate/Consolidation	103/132 (78.0)	99/121 (81.8)	4/11 (36.4)
Bronchopneumonia/Pneumonia	39/132 (29.5)	39/121 (32.2)	0/11 (—)
Pleural effusion	2/132 (1.5)	1/121 (0.8)	1/11 (9.1)
**Chest CT findings (n = 48)^††^**			
Ground glass opacities	21/48 (43.8)	17/40 (42.5)	4/8 (50.0)
Infiltrate/Consolidation	31/48 (64.6)	28/40 (70.0)	3/8 (37.5)
Bronchopneumonia/pneumonia	17/48 (35.4)	15/40 (37.5)	2/8 (25.0)
Pleural effusion	7/48 (14.6)	5/40 (12.5)	2/8 (25.0)
**COVID-19 investigational treatments**			
Received treatment (not mutually exclusive)	52/598 (8.7)	43/272 (15.8)	9/326 (2.8)
Remdesivir	18/598 (3.0)	18/272 (6.6)	0/326 (—)
Azithromycin^§§^	25/598 (4.2)	24/272 (8.9)	1/326 (0.3)
Hydroxychloroquine	21/598 (3.5)	19/272 (7.0)	2/326 (0.6)
Convalescent plasma	9/598 (1.5)	9/272 (3.3)	0/326 (0)
Chloroquine	1/598 (0.2)	1/272 (0.4)	0/326 (0)
Other	17/598 (2.8)	10/272 (3.7)	7/326 (2.2)
**Hospital length of stay, median (IQR), days**	2 (2–3)	3 (2–5)	2 (2–3)
**ICU admission**	44/598 (7.4)	44/272 (16.2)	0/326 (—)
**ICU length of stay, median (IQR), days (n = 41)^¶¶^**	5 (2–13)	5 (2–13)	—
**Interventions**			
Invasive mechanical ventilation***	23/598 (3.8)	23/272 (8.5)	0/326 (—)
BIPAP/CPAP***	3/598 (0.5)	3/272 (1.1)	0/326 (—)
High flow nasal cannula***	5/598 (0.8)	5/272 (1.8)	0/326 (—)
Systemic steroids	34/598 (5.7)	22/272 (8.1)	12/326 (3.7)
Vasopressors	32/598 (5.4)	22/272 (8.1)	10/326 (3.1)
ECMO	2/598 (0.3)	2/272 (0.7)	0/326 (—)
Renal replacement therapy or dialysis	2/598 (0.3)	2/272 (0.7)	0/326 (—)
**New clinical discharge diagnoses (n = 554)^†††^**			
Acute respiratory distress syndrome	15/554 (2.7)	14/251 (5.6)	1/303 (0.3)
Acute respiratory failure	41/554 (7.4)	41/251 (16.3)	0/303 (—)
Pneumonia	75/554 (13.5)	73/251 (29.1)	2/303 (0.7)
Sepsis	21/554 (3.8)	21/251 (8.4)	0/303 (—)
**In-hospital death**	2/598 (0.3)	2/272 (0.7)	0/326 (—)
**Current pregnancy plurality**			
Singleton pregnancy	567/598 (94.8)	253/272 (93.0)	314/326 (96.3)
Multiple pregnancy	14/598 (2.3)	8/272 (2.9)	6/326 (1.8)
Unknown	17/598 (2.8)	11/272 (4.0)	6/326 (1.8)
**Pregnancy-associated conditions (n = 581)^§§§^**			
Gestational diabetes	64/581 (11.0)	31/261 (11.9)	33/320 (10.3)
Hypertensive disorders of pregnancy^¶¶¶^	70/581 (12.0)	33/261 (12.6)	37/320 (11.6)
Intrauterine growth restriction	11/581 (1.9)	4/261 (1.5)	7/320 (2.2)
None	453/581 (78.0)	202/261 (77.4)	251/320 (78.4)
**Pregnancy status at discharge or death**			
Still pregnant	139/598 (23.2)	130/272 (47.8)	9/326 (2.8)
No longer pregnant	458/598 (76.6)	141/272 (51.8)	317/326 (97.2)
Unknown	1/598 (0.2)	1/272 (0.4)	0/326 (—)
**Pregnancy outcomes (n = 458)**			
Live birth****	448/458 (97.8)	134/141 (95.0)	314/317 (99.1)
Term live birth (≥37 wks)^††††^	389/445 (87.4)	103/134 (76.9)	286/311 (92.0)
Pre-term live birth (<37 wks)^††††^	56/445 (12.6)	31/134 (23.1)	25/311 (8.0)
Pregnancy loss^§§§§^	10/458 (2.2)	7/141 (5.0)	3/317 (0.9)
Pregnancy loss at <20 wks’ gestation	4/458 (0.9)	3/141 (2.1)	1/317 (0.3)
Pregnancy loss at ≥20 wks’ gestation	5/458 (1.1)	4/141 (2.8)	1/317 (0.3)
Pregnancy loss at unknown gestational age	1/458 (0.2)	0/141 (–)	1/317 (0.3)
**In-hospital newborn death^¶¶¶¶^**	2/448 (0.4)	2/134 (1.5)	0/314 (—)
**Mode of delivery (n = 458)**			
Vaginal	302/458 (65.9)	79/141 (56.0)	223/317 (70.3)
Cesarean section	151/458 (33.0)	59/141 (41.8)	92/317 (29.0)
Unknown	5/458 (1.1)	3/141 (2.1)	2/317 (0.6)

**Abbreviations:** BIPAP/CPAP = bilevel positive airway pressure/continuous positive airway pressure; COVID-19 = coronavirus disease 2019; CT = computed tomography; ECMO = extracorporeal membrane oxygenation; ICU = intensive care unit; IQR = interquartile range.

* California, Colorado, Connecticut, Georgia, Maryland, Michigan, Minnesota, New Mexico, New York, Ohio, Oregon, Tennessee, and Utah.

^†^ Information not available for those hospitalized before June 2020.

^§^ Does not include gestational diabetes.

^¶^ One or more other underlying conditions, which included blood disorders/hemoglobinopathy (four), immunocompromised condition (two), renal disease (one), and rheumatologic/autoimmune/inflammatory condition (one).

** Among those who had a chest radiograph performed during hospitalization or ≤3 days before the hospital admission.

^††^ Among those who had a chest CT/MRI performed during hospitalization or ≤3 days before the hospital admission.

^§§^ If administered with another COVID-19 investigational treatment.

^¶¶^ Includes women admitted to an ICU with a known ICU length of stay.

*** Highest level of respiratory support for each woman who needed respiratory support.

^†††^ Based on discharge summary diagnoses, for those who had discharge summaries.

^§§§^ Among those with information on pregnancy-associated conditions.

^¶¶¶^ Preeclampsia or gestational hypertension.

**** Number of pregnancies resulting in live birth; might have been a singleton or multiple delivery.

^††††^ Among live births with known gestational age at delivery.

^§§§§^ Pregnancy losses might include spontaneous abortion/miscarriage, therapeutic abortion, or stillbirth.

^¶¶¶¶^ The denominator refers to the 448 pregnancies resulting in live births. These 448 pregnancies resulted in the birth of 457 newborns; nine women had twins. The deaths of two newborns that occurred during the birth hospitalization were indicated on their mothers’ hospital charts.

Among 596 women with COVID-19 whose pregnancy trimester was known, 14 (2.3%), 61 (10.2%), and 521 (87.4%) were hospitalized during the first, second, and third trimesters, respectively. The reason for hospital admission was reported for 324 women: 242 (74.7%) were hospitalized for obstetric indications (including labor and delivery), 61 (18.8%) for COVID-19–related illness, and 21 (6.5%) for other reasons. The most common reason for admission during the first or second pregnancy trimester was COVID-19–related illness (56.8%) and during the third trimester, obstetric indications (81.9%). Among hospitalized pregnant women with COVID-19, 20.6% had at least one underlying medical condition; asthma (8.2%) and hypertension (4.3%) were the most prevalent.

Overall, 272 (45.5%) pregnant women with COVID-19 were symptomatic at the time of hospital admission, and 326 (54.5%) were asymptomatic. Women hospitalized during the first or second trimester were more frequently symptomatic (84.0%) than were those hospitalized during the third trimester (39.9%). Among symptomatic women, the most commonly reported symptoms were fever or chills (59.6%) and cough (59.2%) (Figure 2).

**FIGURE 2:**
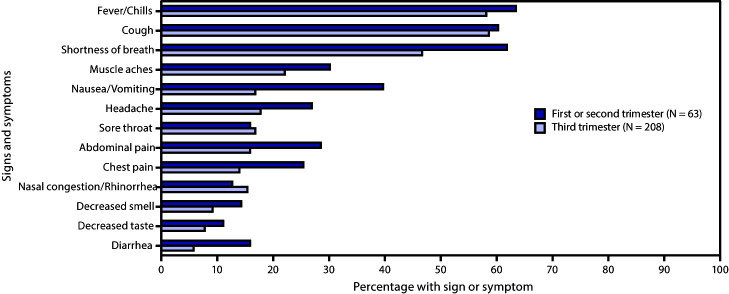
Signs and symptoms* at hospital admission among symptomatic hospitalized pregnant women with COVID-19,^†^ by pregnancy trimester — COVID-NET, 13 states,^§^ March 1–August 22, 2020 **Abbreviations:** COVID-19 = coronavirus disease 2019; COVID-NET = COVID-19-Associated Hospitalization Surveillance Network. * Other signs and symptoms reported on the case report form were upper-respiratory illness/influenza-like illness (11 persons), wheezing (six), hemoptysis/bloody sputum (one), conjunctivitis (one), rash (one), altered mental state (one) and seizure (none). The symptoms decreased smell and decreased taste might not have been ascertained for cases admitted before April 1, 2020, when these symptoms were added as options on the case report form. ^†^ A total of 272 pregnant women with COVID-19 with at least one sign or symptom at the time of hospitalization were identified in COVID-NET. One hospitalized pregnant woman who was symptomatic at admission was not included in this figure because of missing pregnancy trimester. ^§^ California, Colorado, Connecticut, Georgia, Maryland, Michigan, Minnesota, New Mexico, New York, Ohio, Oregon, Tennessee, and Utah.

Among 272 hospitalized symptomatic pregnant women, 44 (16.2%) were admitted to an ICU and 23 (8.5%) required invasive mechanical ventilation. Two (0.7%) deaths were reported among symptomatic women. No asymptomatic women were admitted to an ICU, required invasive mechanical ventilation, or died.

At hospital discharge, 458 women (76.6%) with COVID-19 had completed pregnancies, including 448 (97.8%) that resulted in live births and 10 (2.2%) in pregnancy losses (Figure 1). Pregnancy losses occurred among both symptomatic and asymptomatic hospitalized women with COVID-19 (Table). Four pregnancy losses (0.9% of completed pregnancies) occurred at <20 weeks’ gestation, five (1.1%) at ≥20 weeks’ gestation, and one (0.2%) at unknown gestational age. Among 445 pregnancies resulting in live births with known gestational age at delivery, 87.4% were term births (≥37 weeks’ gestation), and 12.6% were preterm (<37 weeks). Among pregnancies resulting in live births, preterm delivery was reported for 23.1% of symptomatic women and 8.0% of asymptomatic women. Two live-born newborns died during the birth hospitalization (Table); both were born to symptomatic women who required invasive mechanical ventilation.

## Discussion

One in four women aged 15–49 years who had a COVID-19–associated hospitalization during March 1–August 22, 2020 was pregnant, based on a convenience sample from COVID-NET. Approximately one half of pregnant women were asymptomatic at hospital admission. Among symptomatic pregnant women, 16.2% were admitted to an ICU, 8.5% required invasive mechanical ventilation, and two died during COVID-19–associated hospitalizations; none of these outcomes occurred among asymptomatic pregnant women. Among all pregnancies completed during a COVID-19–associated hospitalization, 2.2% resulted in pregnancy losses. Pregnancy losses occurred among both symptomatic and asymptomatic hospitalized women with COVID-19.

Approximately 5% of women of reproductive age in the general population are pregnant at any given time (*1*). The proportion of hospitalized women aged 15–49 years with COVID-19 who were pregnant in this study (26.5%) suggests that pregnant women have disproportionately higher rates of COVID-19–associated hospitalizations compared to nonpregnant women. Although COVID-19 might be more severe in pregnant women, other factors might also explain these higher hospitalization rates. Providers might have a lower threshold for admitting pregnant women for any reason. Some pregnant women with COVID-19 might be admitted solely to give birth. Pregnant women might also have a higher likelihood of being tested for COVID-19 upon admission than do nonpregnant women. Nevertheless, pregnant women account for a substantial proportion of COVID-19–associated hospitalizations among women of reproductive age.

The proportions of hospitalized pregnant women who were Hispanic (42.5%) and Black (26.5%) were higher than the overall proportions of women aged 15–49 years in the COVID-NET catchment area who were Hispanic (15.3%) or Black (19.5%).^§^ Although the racial and ethnic composition of pregnant women in the catchment area is unknown, this report and an earlier study (*1*) suggest that pregnant women who are Hispanic or Black might have disproportionately higher rates of COVID-19–associated hospitalization, compared with those of pregnant women of other races and ethnicities. Long standing inequities in the social determinants of health, such as occupation and housing circumstances that make physical distancing challenging, have put some racial and ethnic minority groups at increased risk for COVID-19–associated illness and death (*4*,*5*). Better understanding of the circumstances under which Hispanic and Black women of reproductive age are exposed to SARS-CoV-2 could inform prevention strategies.

Most pregnant women with COVID-19 in this study were asymptomatic, similar to findings in settings where universal SARS-CoV-2 testing is conducted upon admission to labor and delivery units (*6*). Testing policies based on the presence of symptoms might miss many SARS-CoV-2 infections during pregnancy. Early identification of COVID-19 among hospitalized pregnant women can help ensure that health care providers use appropriate personal protective equipment and limit visitors to those essential for patients’ well-being and care.^¶^

The overall proportion of pregnant women with COVID-19 admitted to an ICU (7.4%) was similar to that observed in two European studies (*7*,*8*); however, 16.2% of symptomatic pregnant women in this study were admitted to an ICU, indicating that outcomes might be more severe among pregnant women admitted with acute illness than among those admitted for obstetric indications alone.

Although the preterm delivery rate in the study catchment area during the surveillance period is unknown, the prevalence of preterm delivery among live births during COVID-19–associated hospitalizations (12.6%) was higher than that observed in the general U.S. population in 2018 (10.0%) (*9*). In this study, preterm births occurred approximately three times more frequently in symptomatic pregnant women than in those who were asymptomatic. Preterm newborns might be at increased risk for severe COVID-19 illness, and preventive measures, such as encouraging caretakers to wear a mask and practice hand hygiene, should be emphasized to minimize possible transmission.**

Birth outcomes in this analysis were limited to pregnancies completed during a COVID-19–associated hospitalization. COVID-NET only captured medically attended pregnancy losses and likely underestimates the percentage of pregnancy losses that occur among women with COVID-19. Further prospective data on birth outcomes among women infected during all pregnancy trimesters is needed. CDC is collaborating with state and local health departments to conduct detailed surveillance of pregnant women with COVID-19 and their infants.^††^

The findings in this report are subject to at least six limitations. First, at the time of analysis, chart abstractions were ongoing and completed for a convenience sample of 29.4% of women aged 15–49 years. Thus, the estimated proportion of hospitalized women with COVID-19 who were pregnant might be biased, because pregnancy status was not yet ascertained for women without completed chart review. Second, pregnant women included in this analysis might not be representative of all pregnant women within the catchment area. Third, COVID-19 cases might have been missed because of testing practices and test availability, which likely varied across time and facilities. Fourth, the reason for hospital admission was unavailable for 45.8% of women, limiting the ability to distinguish between admissions solely for labor and delivery and those for COVID-19–related illness. Fifth, information on obesity as an underlying prepregnancy condition was not available, so this underlying health condition could not be described. Finally, information on maternal and newborn mortality was only obtained from the maternal medical chart and did not capture outcomes occurring beyond the COVID-19–associated hospitalization.

Severe illness and adverse birth outcomes were observed among hospitalized pregnant women with COVID-19. These findings highlight the importance of preventing and identifying COVID-19 in pregnant women. Pregnant women should avoid close contact with persons with confirmed or suspected COVID-19, maintain 6 feet of distance from nonhousehold members, and take general COVID-19 preventive measures, including wearing masks and practicing hand hygiene.^§§^ CDC recommends testing newborns born to mothers with COVID-19, isolation of mothers with COVID-19 and their newborns from other hospitalized mothers and newborns, and infection prevention measures for persons caring for newborns who might be exposed to SARS-CoV-2.** Continued surveillance for COVID-19 in pregnant women is important to understand and improve health outcomes for mothers and newborns.

**Summary**

**What is already known about this topic?**

Information on the clinical characteristics and birth outcomes of hospitalized U.S. pregnant women with COVID-19 is limited.

**What is added by this report?**

Among 598 hospitalized pregnant women with COVID-19, 55% were asymptomatic at admission. Severe illness occurred among symptomatic pregnant women, including intensive care unit admissions (16%), mechanical ventilation (8%), and death (1%). Pregnancy losses occurred for 2% of pregnancies completed during COVID-19-associated hospitalizations and were experienced by both symptomatic and asymptomatic women.

**What are the implications for public health practice?**

Pregnant women and health care providers should be aware of potential risks for severe COVID-19, including adverse pregnancy outcomes. Identifying COVID-19 during birth hospitalizations is important to guide preventive measures to protect pregnant women, parents, newborns, other patients, and hospital personnel.

## Footnotes

* Counties in COVID-NET surveillance: *California* (Alameda, Contra Costa, and San Francisco); *Colorado* (Adams, Arapahoe, Denver, Douglas, and Jefferson); *Connecticut* (Middlesex and New Haven); *Georgia* (Clayton, Cobb, DeKalb, Douglas, Fulton, Gwinnett, Newton, and Rockdale); *Iowa* (one county); *Maryland* (Allegany, Anne Arundel, Baltimore, Baltimore City, Calvert, Caroline, Carroll, Cecil, Charles, Dorchester, Frederick, Garrett, Harford, Howard, Kent, Montgomery, Prince George’s, Queen Anne’s, St. Mary’s, Somerset, Talbot, Washington, Wicomico, and Worcester); *Michigan* (Clinton, Eaton, Genesee, Ingham, and Washtenaw); *Minnesota* (Anoka, Carver, Dakota, Hennepin, Ramsey, Scott, and Washington); *New Mexico* (Bernalillo, Chaves, Doña Ann, Grant, Luna, San Juan, and Santa Fe); *New York* (Albany, Columbia, Genesee, Greene, Livingston, Monroe, Montgomery, Ontario, Orleans, Rensselaer, Saratoga, Schenectady, Schoharie, Wayne, and Yates); *Ohio* (Delaware, Fairfield, Franklin, Hocking, Licking, Madison, Morrow, Perry, Pickaway, and Union); *Oregon* (Clackamas, Multnomah, and Washington); *Tennessee* (Cheatham, Davidson, Dickson, Robertson, Rutherford, Sumner, Williamson, and Wilson); and *Utah* (Salt Lake).

† 45 C.F.R. part 46.102(l)(2), 21 C.F.R. part 56; 42 U.S.C. Sect. 241(d); 5 U.S.C. Sect. 552a; 44 U.S.C. Sect. 3501 et seq.

§ The distribution of race/ethnicity among the COVID-NET catchment area was calculated using 2019 vintage NCHS bridged-race population estimates. https://www.cdc.gov/nchs/nvss/bridged_race.htm.

¶ https://www.cdc.gov/coronavirus/2019-ncov/hcp/inpatient-obstetric-healthcare-guidance.html.

** https://www.cdc.gov/coronavirus/2019-ncov/hcp/caring-for-newborns.html.

†† https://www.cdc.gov/coronavirus/2019-ncov/cases-updates/special-populations/pregnancy-data-on-covid-19.html; https://www.cdc.gov/ncbddd/aboutus/pregnancy/emerging-threats.html.

§§ https://www.cdc.gov/coronavirus/2019-ncov/need-extra-precautions/pregnancy-breastfeeding.html.
